# Subsyndromal Depressive Symptoms in Bipolar Disorder: Associations with Functioning, Internalized Stigma, Impulsivity, and Anxiety

**DOI:** 10.3390/brainsci16090943

**Published:** 2026-09-03

**Authors:** Hazel Demiröz Öztürk, Medine Gıynaş Ayhan, İkbal İnanlı

**Affiliations:** Department of Psychiatry, Beyhekim Training and Research Hospital, University of Health Sciences, Konya 42060, Türkiye; drmedineayhan@gmail.com (M.G.A.); ikbalcivi@yahoo.com (İ.İ.)

**Keywords:** bipolar disorder, subsyndromal symptoms, functioning, impulsivity, internalized stigma, anxiety

## Abstract

**Highlights:**

**What are the main findings?**

- Patients with subsyndromal depressive symptoms showed poorer functioning and higher levels of impulsivity, anxiety, and internalized stigma than euthymic patients.
- Within the subsyndromal depressive group, depressive symptom severity was associated with specific domains of functioning, impulsivity, internalized stigma, and anxiety.

**What are the implications of the main findings?**

- Subsyndromal depressive symptoms may be associated with clinically relevant psychosocial differences during inter-episode periods in bipolar I disorder.
- Considering depressive symptom severity dimensionally alongside categorical assessment may provide a more comprehensive characterization of subsyndromal depressive symptoms.

**Abstract:**

**Backgrounds/Objectives**: Subsyndromal depressive symptoms are common during inter-episode periods in bipolar disorder and have been associated with functional impairment. This study aimed to examine their clinical and psychosocial correlates across multiple domains and to evaluate depressive symptom severity from both categorical and dimensional perspectives. **Methods**: This cross-sectional study included 133 outpatients with bipolar I disorder who had experienced no syndromal mood episode during the preceding six months. Patients were classified as having subsyndromal depressive symptoms (*n* = 61; HDRS 8–14 and YMRS < 8) or as euthymic (*n* = 72; HDRS < 8 and YMRS < 8). Clinical characteristics, functioning, impulsivity, internalized stigma, and anxiety were assessed. **Results**: The subsyndromal depressive group had higher total and depressive episode counts and more frequent histories of antidepressant-induced manic switch and suicide attempt than the euthymic group (all *p* < 0.05). They also showed lower functioning and higher impulsivity, internalized stigma, and anxiety (all *p* < 0.05). Within the subsyndromal depressive group, depressive symptom severity was associated with the number of depressive episodes and specific domains of functioning, impulsivity, internalized stigma, and anxiety. **Conclusions**: Subsyndromal depressive symptoms were associated with clinically relevant differences across multiple clinical and psychosocial domains during inter-episode periods in bipolar I disorder. The associations observed across specific domains also support considering depressive symptom severity dimensionally alongside categorical assessment.

## 1. Introduction

Bipolar disorder is a chronic mood disorder characterized by recurrent manic and depressive episodes and marked functional impairment [1,2]. Although it is classically defined by discrete mood episodes, a substantial proportion of patients continue to experience a significant burden of psychiatric symptoms even during inter-episode periods that are clinically considered euthymic [3]. These symptoms, while not meeting full diagnostic criteria, may still have clinically meaningful consequences and are commonly referred to as subsyndromal symptoms [2]. Although there is no universally accepted definition of subsyndromal symptoms, the International Society for Bipolar Disorders (ISBD) has proposed operational criteria, defining subsyndromal depression as a score of 8–14 on the Hamilton Depression Rating Scale (HDRS) and subsyndromal mania as a score of 8–14 on the Young Mania Rating Scale (YMRS) [4]. In a longitudinal cohort study using ISBD criteria over approximately five years, subsyndromal symptoms were identified in more than one-fifth of patients [5]. Furthermore, several studies have demonstrated that subsyndromal symptoms are present for nearly half of the lifetime of individuals with bipolar disorder [6]. These findings suggest that euthymia in bipolar disorder may not represent a truly symptom-free state, but rather a clinically heterogeneous condition.

The persistence of subsyndromal symptoms has been shown to have significant adverse effects on psychosocial functioning and quality of life, even in patients considered clinically stable. In particular, subsyndromal depression has been associated with impairments in occupational, domestic, social, and cognitive domains, as well as reduced quality of life [2]. Moreover, subsyndromal symptoms have been reported to be associated with an increased frequency of mood episodes, thereby representing a potential risk factor for a more chronic course of illness [7]. Subsyndromal mood symptoms that persist despite treatment may impose a substantial burden on patients’ daily functioning, underscoring their clinical relevance beyond the occurrence of acute mood episodes [8].

In euthymic patients with bipolar disorder, it has been previously shown that depressive symptom severity, even when below the diagnostic threshold, is associated with functional outcomes [9]. As the severity of depressive symptoms increases, greater impairment in functioning is observed [9], and significant associations have also been reported with psychosocial variables such as impulsivity, anxiety, and internalized stigma [10,11,12,13]. Thus, the association between subsyndromal depressive symptoms and functional impairment is already well established. However, studies simultaneously evaluating multiple psychosocial constructs within the same sample, particularly at the level of specific subdomains, remain limited. Furthermore, examining subsyndromal depressive symptoms from both categorical and dimensional perspectives may provide a more detailed characterization of their clinical and psychosocial correlates.

Accordingly, this study aimed to examine the clinical and psychosocial correlates of subsyndromal depressive symptoms in outpatients with bipolar I disorder. Patients in the subsyndromal depressive group were compared with those in the euthymic group in terms of sociodemographic and clinical characteristics, functioning, impulsivity, internalized stigma, and anxiety. In addition, within the subsyndromal depressive group, associations between depressive symptom severity (HDRS scores) and these psychosocial constructs and their subdomains were examined. This approach enabled the simultaneous evaluation of multiple psychosocial domains while incorporating both categorical group comparisons and a dimensional assessment of depressive symptom severity.

## 2. Materials and Methods

### 2.1. Study Design and Ethical Approval

This cross-sectional observational study was designed to investigate the associations of subsyndromal depressive symptoms with clinical characteristics, functioning, impulsivity, internalized stigma, and anxiety in patients with bipolar I disorder. The study was conducted between October 2023 and March 2024 at the outpatient psychiatry clinic of Beyhekim Training and Research Hospital, University of Health Sciences, Konya, Türkiye.

The study protocol was approved by the Ethics Committee of the Faculty of Medicine, KTO Karatay University (decision no. E-41901325-200-69169; 26 September 2023). The study was conducted in accordance with the principles of the Declaration of Helsinki. All participants were informed about the study and provided written informed consent prior to participation.

An a priori power analysis was performed using G*Power software (version 3.1; Heinrich Heine University Düsseldorf, Düsseldorf, Germany). Assuming a two-sided significance level of α = 0.05, a statistical power of 1 − β = 0.95, and a medium effect size (Cohen’s d = 0.50), the minimum required sample size was calculated as 42 participants per group, corresponding to a total sample of at least 84 participants.

### 2.2. Participants and Eligibility Criteria

Patients aged 18–65 years who attended the outpatient psychiatry clinic during the study period and had a diagnosis of bipolar I disorder according to the Diagnostic and Statistical Manual of Mental Disorders, Fifth Edition (DSM-5), were assessed for eligibility. The diagnosis of bipolar I disorder was confirmed by a psychiatrist based on clinical interviews and review of hospital/electronic medical records.

The inclusion criteria were age between 18 and 65 years, a diagnosis of bipolar I disorder according to DSM-5 criteria, willingness to participate, provision of written informed consent, literacy, and the absence of severe visual or auditory impairment that could interfere with study assessments. To minimize the possibility that current clinical symptoms represented the resolution of a recent mood episode, patients were also required to have experienced no syndromal depressive, manic, or mixed mood episode during the six months preceding the study assessment.

The exclusion criteria were the presence of any comorbid psychiatric disorder meeting full diagnostic criteria, intellectual disability, a history of alcohol or substance use disorder, severe physical illness, a syndromal mood episode or psychiatric hospitalization during the preceding six months, concomitant subsyndromal manic symptoms at the time of assessment, and use of systemic medications with the potential to influence mood symptoms, such as corticosteroids or interferons. Patients with HDRS or YMRS scores > 14 were also excluded because their symptom severity was considered incompatible with the subsyndromal depressive or euthymic clinical states investigated in this study.

Psychiatric comorbidities, history of alcohol or substance use disorder, previous mood episodes, psychiatric hospitalizations, and other exclusion criteria were assessed by a psychiatrist based on clinical interviews and review of hospital/electronic medical records.

### 2.3. Participant Selection and Definition of Study Groups

A total of 162 patients were assessed for eligibility. Of these, 29 were excluded because of unwillingness to participate (*n* = 6), illiteracy (*n* = 3), an HDRS score > 14 (*n* = 2), severe chronic physical illness (*n* = 7), intellectual disability (*n* = 1), a history of alcohol or substance use disorder (*n* = 7), or concomitant subsyndromal manic symptoms (*n* = 5). Two patients met both the alcohol/substance use disorder history and concomitant subsyndromal manic symptom exclusion criteria; therefore, the total number of unique patients excluded was 29.

The final analytic sample comprised 133 patients. At the study assessment, depressive symptom severity was evaluated using the Hamilton Depression Rating Scale (HDRS), and manic symptom severity was evaluated using the Young Mania Rating Scale (YMRS), both administered by a clinician. Patients with HDRS scores of 8–14 and YMRS scores < 8 were classified as the subsyndromal depressive group, whereas patients with both HDRS and YMRS scores < 8 were classified as the euthymic group. Accordingly, the final analytic sample consisted of 61 patients with subsyndromal depressive and 72 euthymic group.

Clinical interviews and review of medical records confirmed that none of the patients included in the final cohort had experienced a syndromal depressive, manic, or mixed mood episode during the six months preceding the study assessment. The six-month episode-free interval was used to minimize the possibility that subsyndromal depressive symptoms identified at assessment reflected the resolution of a recent syndromal mood episode.

### 2.4. Sociodemographic, Clinical, and Treatment Characteristics

Sociodemographic, clinical, and treatment characteristics were recorded using a structured data collection form developed by the investigators for the present study. The form was completed by a psychiatrist based on clinical interviews with the patient, information obtained from an accompanying family member when necessary, and review of hospital/electronic medical records.

Sociodemographic variables included age, sex, marital status, parental status, and employment status. Clinical variables included age at illness onset, illness duration, total number of mood episodes, numbers of depressive and manic episodes, lifetime number of psychiatric hospitalizations, polarity of the first mood episode, family history of bipolar disorder, history of antidepressant-induced manic switch, lifetime history of suicide attempt, history of electroconvulsive therapy (ECT), and history of repetitive transcranial magnetic stimulation (rTMS).

A history of antidepressant-induced manic switch was determined retrospectively from hospital/electronic medical records and was not prospectively assessed as part of the present study.

Pharmacological treatments being used at the time of study assessment were also recorded from hospital/electronic medical records. Treatment regimens were classified into five categories: mood stabilizer (MS) monotherapy, antipsychotic (AP) monotherapy, MS + AP, AP + antidepressant (AD), and MS + AP + AD. In addition, histories of ECT and rTMS and receipt of ongoing psychotherapy were assessed. None of the participants were receiving ongoing psychotherapy at the time of the study assessment. No specific pharmacological treatment regimen was required for study inclusion, and no changes or interventions were made to participants’ ongoing treatments as part of the study.

### 2.5. Assessment of Functioning, Impulsivity, Internalized Stigma, and Anxiety

Following the clinical evaluation, self-report scales were administered to assess psychosocial functioning, impulsivity, and internalized stigma. The instruments used included the Bipolar Disorder Functioning Scale (BDFS), the Barratt Impulsiveness Scale (BIS-11), the Internalized Stigma of Mental Illness Scale (ISMI), and the Beck Anxiety Inventory (BAI).

The BDFS was developed to evaluate multiple domains of functioning in bipolar disorder and provides a comprehensive assessment of patients’ daily activities, interpersonal relationships, and social roles. Total scores range from 52 to 156, with higher scores indicating better levels of functioning [14].

The BIS-11 is a widely used measure that assesses impulsivity across attentional, motor, and non-planning domains. The scale consists of 30 items, with total scores ranging from 30 to 120. Higher scores indicate greater levels of impulsivity [15].

The ISMI is a self-report instrument used to assess the level of internalized stigma related to mental illness. The scale consists of 29 items and includes five subdomains: alienation, stereotype endorsement, perceived discrimination, social withdrawal, and stigma resistance. Total scores range from 4 to 91, with higher scores indicating greater levels of internalized stigma [16].

The Beck Anxiety Inventory (BAI) is a 21-item self-report instrument that assesses the severity of anxiety symptoms, including both subjective and somatic manifestations. Total scores range from 0 to 63, with higher scores indicating greater anxiety severity [17]. All instruments have previously been validated and shown to be reliable in Turkish populations [14,15,16,17]. When necessary, participants who experienced difficulty understanding the items were provided with clarifying guidance by the researcher.

### 2.6. Statistical Analysis

Statistical analyses were performed using IBM SPSS Statistics for Windows, Version 26.0 (IBM Corp., Armonk, NY, USA). The distribution of continuous variables was assessed using the Shapiro–Wilk test. Descriptive statistics were presented as the mean ± standard deviation for normally distributed continuous variables and as the median (interquartile range: 1st–3rd quartiles) for non-normally distributed variables, while categorical variables were expressed as frequencies and percentages (%). Comparisons between the subsyndromal depressive group and the euthymic group were conducted using the independent samples *t*-test for normally distributed continuous variables and the Mann–Whitney U test for non-normally distributed variables. The chi-square test was used for comparisons of categorical variables. In the subsyndromal depressive group, associations between depressive symptom severity and clinical as well as psychosocial variables were evaluated using Spearman’s correlation analysis. A *p*-value of <0.05 was considered statistically significant in all analyses. Given the exploratory nature of the study, no correction for multiple comparisons was applied; therefore, the findings should be interpreted with caution.

## 3. Results

A total of 133 patients with bipolar I disorder were analyzed, of whom 61 were classified as the subsyndromal depressive group and 72 as the euthymic group. The sociodemographic and clinical characteristics of the groups, together with measures of functioning, internalized stigma, impulsivity, and anxiety, are presented in Table 1, Table 2 and Table 3. No significant differences were observed between the subsyndromal depressive and euthymic groups in terms of age, sex, marital status, parenthood, or employment status (all *p* > 0.05; Table 1).

Regarding clinical variables (Table 1), the total number of mood episodes and the number of depressive episodes were significantly higher in the subsyndromal depressive group compared to the euthymic group (*p* = 0.039 and *p* = 0.003, respectively). A history of antidepressant-induced manic switch and a lifetime history of suicide attempt were also more frequent in the subsyndromal depressive group (*p* = 0.010 and *p* = 0.009, respectively). No significant differences were observed between the groups regarding age at illness onset, duration of illness, number of manic episodes, lifetime number of psychiatric hospitalizations, family history of bipolar disorder, history of ECT, or current pharmacological treatment distribution (all *p* > 0.05).

Regarding functioning, internalized stigma, impulsivity, and anxiety measures (Table 2), BDFS total scores were lower in the subsyndromal depressive group compared to the euthymic group (*p* < 0.001).

Analysis of the subdomains revealed significantly lower levels of functioning in the subsyndromal depressive group across sexual functioning, domestic relationships, emotional functioning, cognitive functioning, perceived stigma, and social withdrawal (all *p* < 0.05). For internalized stigma, all subscale scores of the ISMI, except for stigma resistance, were significantly higher in the subsyndromal depressive group (all *p* < 0.05). Regarding impulsivity, both the total score and all subscale scores of the BIS-11 were significantly higher in the subsyndromal depressive group compared to the euthymic group (all *p* < 0.05). BAI scores were significantly higher in the subsyndromal depressive group (*p* < 0.001).

The associations between depressive symptom severity and clinical as well as psychosocial variables in patients with subsyndromal symptoms are presented in Table 3.

A significant positive correlation was observed between HDRS total scores and the number of depressive episodes (r = 0.281, *p* = 0.020). No significant correlations were observed between HDRS scores and age at illness onset, duration of illness, total number of episodes, or number of hospitalizations (all *p* > 0.05). In terms of psychosocial functioning, negative correlations were found between HDRS scores and the sexual functioning (r = −0.341, *p* = 0.005) and domestic relationships (r = −0.289, *p* = 0.017) subdomains of the BDFS. No significant associations were observed between HDRS scores and the total BDFS score or its other subdomains. For impulsivity, a positive correlation was found between HDRS scores and the attentional impulsivity subscale (r = 0.315, *p* = 0.009). No associations were observed with the other subscales or the total BIS-11 score. In terms of internalized stigma, a positive correlation was found between HDRS scores and the “alienation” subscale of the ISMI (r = 0.249, *p* = 0.047). No significant associations were observed with the other subscales or the total ISMI score. A positive correlation was also observed between anxiety levels and HDRS scores (r = 0.465, *p* < 0.001).

## 4. Discussion

In this study, we examined the clinical and psychosocial correlates of subsyndromal depressive symptoms in patients with bipolar I disorder during the inter-episode period. Rather than focusing solely on the previously established association between subsyndromal depressive symptoms and functional impairment, the present study simultaneously evaluated functioning, impulsivity, internalized stigma, and anxiety, including their specific subdomains where applicable, and incorporated both categorical group comparisons and a dimensional assessment of depressive symptom severity. Patients in the subsyndromal depressive group exhibited poorer functioning and higher levels of impulsivity, internalized stigma, and anxiety compared with the euthymic group. Additionally, within the subsyndromal depressive group, depressive symptom severity was associated with specific domains of functioning, attentional impulsivity, internalized stigma, and anxiety.

With regard to sociodemographic and clinical characteristics, patients in the subsyndromal depressive group and the euthymic group were comparable in terms of age, sex, marital status, duration of education, employment status, and place of residence. This suggests that the observed differences are unlikely to be substantially influenced by these variables. Consistent with previous literature indicating that euthymia does not represent a homogeneous clinical state [18,19], our findings suggest that the presence of subsyndromal depressive symptoms is associated with clinically meaningful differences. In particular, the higher total number of episodes and greater burden of depressive episodes in the subsyndromal depressive group suggest a more recurrent course of illness. This is in line with previous studies indicating that residual symptoms may adversely affect the long-term course of bipolar disorder [20]. Furthermore, the higher rate of antidepressant-induced manic switch in the subsyndromal depressive group may indicate a more complex clinical course and treatment history. A lifetime history of suicide attempt was also more frequent in the subsyndromal depressive group than in the euthymic group. Although clinically relevant, this finding should be interpreted as an association; given the cross-sectional design and the lifetime nature of the variable, it does not indicate that current subsyndromal depressive symptoms increase the risk of suicide attempts. Overall, these findings suggest that subsyndromal depressive symptoms may be associated with a more recurrent and clinically complex course of illness.

The simultaneous assessment of multiple psychosocial constructs revealed differences across functioning, impulsivity, internalized stigma, and anxiety between the two groups. Patients in the subsyndromal depressive group demonstrated higher levels of anxiety, impulsivity, and internalized stigma, as well as lower levels of functioning compared with the euthymic group. While the association between subsyndromal depressive symptoms and functional impairment is consistent with previous literature [18,21,22], the present findings extend this evidence by demonstrating differences across several psychosocial constructs within the same sample and by characterizing specific subdomains of impairment. In terms of functioning, differences were observed across multiple domains, including emotional, cognitive, domestic, and sexual functioning.

The higher levels of impulsivity observed in the subsyndromal depressive group suggest that elevated impulsivity may also be observed during inter-episode periods in patients with subsyndromal depressive symptoms. This finding is consistent with previous studies indicating elevated impulsivity in individuals with bipolar disorder even during remission [23]. In line with this, the higher levels of anxiety in the subsyndromal depressive group suggest that the inter-episode period may not be entirely asymptomatic from a clinical perspective. These findings suggest that anxiety symptoms may also be associated with subsyndromal depressive symptoms during inter-episode periods, consistent with the existing literature [24,25]. The higher levels of internalized stigma in the subsyndromal depressive group further suggest that these symptoms may influence not only clinical outcomes but also individuals’ self-perception and social identity. In particular, increases observed in subdomains such as alienation and social withdrawal indicate that subsyndromal depressive symptoms may also affect individuals’ interactions with their social environment. This finding is consistent with previous studies demonstrating a positive association between subsyndromal symptom severity and levels of internalized stigma [26].

In the subsyndromal depressive group, depressive symptom severity was examined as a continuous variable based on HDRS scores. No significant relationships were found between HDRS scores and the sociodemographic variables examined in the subsyndromal depressive group. Although not statistically significant, higher HDRS scores were observed in patients with a history of antidepressant-induced manic switch and suicide attempts, which may indicate a potential association with clinical instability. These exploratory findings should be interpreted cautiously and warrant further investigation in larger longitudinal studies to clarify whether depressive symptom severity during inter-episode periods is associated with subsequent mood instability and suicidal outcomes. Regarding illness characteristics, HDRS scores were positively correlated with the number of depressive episodes, suggesting that the cumulative burden of depressive episodes may persist during inter-episode periods and be associated with subsyndromal depressive symptom severity. Consistent with this, previous studies have reported an association between depressive episode burden and residual or subsyndromal depressive symptoms [27]. In contrast, the absence of significant associations with variables such as illness duration, number of manic episodes, and number of hospitalizations suggests that subsyndromal depressive symptom severity may not be uniformly related to all clinical dimensions of the disorder.

The association between depressive symptom severity and functional domains suggests that subsyndromal depressive symptoms may be linked to impairments in concrete aspects of daily life. Significant correlations with domestic and sexual functioning indicate that increasing symptom severity may be associated with difficulties in maintaining everyday activities and interpersonal functioning. Domestic functioning, which includes instrumental activities of daily living such as taking responsibility, maintaining planned tasks, and fulfilling household roles, may be particularly vulnerable. Consistent with previous studies reporting associations between depressive symptom burden and functional independence [28], our findings suggest that these impairments may persist even during inter-episode periods. From a clinical perspective, subtle functional difficulties may be overlooked in patients considered clinically stable. These findings underscore the importance of assessing not only the presence of symptoms but also the severity of subsyndromal depressive symptoms when evaluating functioning.

With respect to sexual functioning, the association between HDRS scores and sexual functioning suggests that subsyndromal depressive symptoms may also affect more personal domains of functioning. Although it is well established that sexual desire tends to increase during manic episodes and decrease during depressive episodes in bipolar disorder [29], data on sexual functioning during remission or subsyndromal states remain limited. Previous studies have reported that sexual dysfunction may persist even in individuals considered euthymic but exhibiting residual symptoms [30]. Our findings are consistent with this literature and further support an association between depressive symptom severity and sexual functioning during inter-episode periods. However, sexual functioning in bipolar disorder is multifactorial and may also be influenced by pharmacological treatment and treatment-related effects. Although the distribution of current pharmacological treatment categories did not differ significantly between the groups in the present study, the effects of individual medications, doses, prolactin-related effects, and sedation were not separately assessed. Therefore, the observed association between depressive symptom severity and sexual functioning should be interpreted cautiously and regarded as hypothesis-generating rather than as evidence of an independent relationship. From a clinical perspective, although sexual functioning is often overlooked in the follow-up of bipolar disorder, it may warrant greater clinical attention given its close association with quality of life and subjective well-being.

Associations between depressive symptom severity and psychosocial dimensions were also examined. A significant relationship was observed between HDRS scores and attentional impulsivity. This finding suggests that impulsivity may extend beyond acute mood episodes and vary in parallel with depressive symptom severity during inter-episode periods. Although increased levels of impulsivity were observed across all subdomains in the group comparisons, the association with symptom severity was limited to the attentional component, suggesting that this dimension may be particularly sensitive to depressive symptoms. Previous studies have reported that attentional impulsivity may be associated with depressive cognitive features such as anhedonia and hopelessness [11], as well as with suicidal tendencies [31,32,33]. This finding suggests that attentional impulsivity may extend beyond acute mood episodes and be associated with depressive symptom severity during inter-episode periods. The emergence of this relationship in the subsyndromal depressive group raises the possibility that certain cognitive processes remain affected during inter-episode periods. From a clinical perspective, increased attentional impulsivity may adversely affect decision-making, daily planning, and behavioral regulation. Accordingly, it may also be considered one of the indirect pathways through which subsyndromal depressive symptoms impact overall functioning.

When examined in terms of internalized stigma, a significant positive association was observed between HDRS scores and the “alienation” subscale. This finding suggests that increasing depressive symptom severity may be associated with greater feelings of social disconnection or reduced sense of belonging. The association was observed not across all stigma subdomains but specifically with the alienation dimension, suggesting that depressive symptom severity may be more closely related to individuals’ subjective social experiences and self-perception. Although previous studies have demonstrated a relationship between depressive symptom severity and internalized stigma, studies examining stigma at the subdomain level remain limited [26,34,35]. These findings also suggest that internalized stigma is not a homogeneous construct and that the alienation dimension, in particular, may be more strongly associated with depressive symptom severity. The absence of associations with other stigma subdomains may indicate that these dimensions are less directly related to current depressive symptom severity.

Regarding anxiety symptoms, the positive correlation observed between HDRS and BAI scores suggests that depressive symptom severity and anxiety levels may co-occur even during inter-episode periods. Previous studies have reported that comorbid anxiety symptoms in bipolar disorder may be associated with a more unfavorable clinical course [21,36,37]. Our findings further suggest that anxiety in bipolar disorder may be conceptualized not only as a comorbid condition but also as a dimensional feature associated with depressive symptom severity. Moreover, the observed association between anxiety and depressive symptom severity suggests that these domains may reflect closely related processes. From a clinical perspective, the persistence of anxiety symptoms even during inter-episode periods should be taken into consideration, as they may increase patients’ subjective distress and further exacerbate impairments in functioning.

Several limitations should be considered when interpreting these findings. First, due to its cross-sectional design, causal inferences regarding the directionality of the observed associations cannot be established. Although the absence of a syndromal mood episode during the six months preceding the study assessment reduced the likelihood that the observed subsyndromal depressive symptoms represented the resolution of a recent episode, their longitudinal persistence could not be established because symptom severity was assessed cross-sectionally. Therefore, transient fluctuations in depressive symptoms during the inter-episode period cannot be entirely excluded. Longitudinal studies using repeated symptom assessments are needed to distinguish persistent subsyndromal depressive symptoms from transient symptom fluctuations. Second, the study was conducted in a single center and included only outpatients, which may limit the generalizability of the findings. Third, psychiatric comorbidities were assessed based on clinical evaluation and medical records; therefore, the absence of structured diagnostic interviews may have resulted in under-recognition of comorbid disorders in remission. Moreover, the exclusion of patients with current psychiatric comorbidities may have resulted in a more clinically selected sample, further limiting the generalizability of the findings to the broader population of patients with bipolar I disorder. Fourth, the use of self-report measures for the assessment of psychosocial variables may have introduced response bias. In addition, because the groups differed in several illness-course characteristics and the main psychosocial comparisons were not adjusted for these differences, caution is warranted when interpreting the observed associations as being independent of illness-course severity. Fifth, although current pharmacological treatment regimens were evaluated in the revised analyses, the potential effects of individual medications, doses, treatment duration, and treatment-related adverse effects were not examined separately and may have influenced some of the outcomes. This limitation is particularly relevant to sexual functioning, which may be influenced by antidepressant- and antipsychotic-related adverse effects, including sexual dysfunction, sedation, and prolactin-related effects. Finally, the definition of subsyndromal depressive symptoms based on scale cut-off scores may limit the interpretation of the findings. Despite these limitations, the study also has several strengths. The principal contribution of the present study lies in the simultaneous assessment of multiple psychosocial constructs within the same sample, rather than in establishing the already recognized association between subsyndromal depressive symptoms and functional impairment. The combined categorical and dimensional approach allowed comparisons between the subsyndromal depressive and euthymic groups while also examining associations between depressive symptom severity and clinical and psychosocial variables within the subsyndromal depressive group. In addition, the assessment of functioning, impulsivity, internalized stigma, and anxiety at the subdomain level where applicable provided a more detailed characterization of the psychosocial correlates of subsyndromal depressive symptoms.

## 5. Conclusions

This study demonstrates that subsyndromal depressive symptoms in patients with bipolar I disorder may be associated with meaningful differences in clinical and psychosocial domains. The finding that patients in the subsyndromal depressive group exhibit more pronounced clinical characteristics and psychosocial differences compared to the euthymic group suggests that patients with bipolar I disorder may exhibit clinically meaningful heterogeneity even outside syndromal mood episodes. Furthermore, within the subsyndromal depressive group, depressive symptom severity was associated with specific domains of functioning, impulsivity, internalized stigma, and anxiety, supporting the relevance of a dimensional approach that considers symptom severity in addition to its presence. These findings suggest that subsyndromal depressive symptoms, which may be overlooked during inter-episode periods, should be considered in the clinical follow-up of bipolar I disorder. Longitudinal studies are needed to further clarify the direction and potential clinical implications of these associations.

## Figures and Tables

**Table 1:** Comparison of sociodemographic and clinical characteristics of the subsyndromal depressive group and euthymic groups.

	Subsyndromal Depressive (*n* = 61)	Euthymic (*n* = 72)	*p*
Sociodemographic Characteristics			
Age, years; median (IQR)	37 (30–47)	36 (29–46)	0.587
Married, *n* (%)	30 (49.2)	24 (33.3)	0.201
Parenthood, *n* (%)	32 (52.4)	31 (43.0)	0.314
Employed, *n* (%)	27 (44.3)	38 (52.8)	0.154
Sex (female), *n* (%)	25 (40.9)	29 (40.3)	0.919
Clinical Characteristics			
Age at onset, median (IQR)	21 (18–28)	21 (18–29)	0.441
Illness duration, years, median (IQR)	13 (8–20)	11 (7–21)	0.501
Total number of episodes, median (IQR)	4 (3–7)	3 (2–6)	0.039
Number of depressive episodes, median (IQR)	1 (0–2)	0 (0–1)	0.003
Number of manic episodes, median (IQR)	3 (1–5)	3 (1–4)	0.891
Lifetime number of hospitalizations, median (IQR)	3 (1–5)	2 (1–4)	0.202
Manic first episode, *n* (%)	34 (55.7)	58 (80.5)	0.011
Family history of bipolar disorder, *n* (%)	18 (29.5)	16 (22.2)	0.421
History of ECT, *n* (%)	18 (29.45)	18 (25.0)	0.671
History of rTMS, *n* (%)	0 (0)	0 (0)	-
Antidepressant-induced manic switch, *n* (%)	14 (22.9)	5 (6.9)	0.010
History of suicide attempt, *n* (%)	19 (31.1)	9 (12.5)	0.009
Current pharmacological treatment, *n* (%)			0.885
Mood stabilizer only	9 (14.8)	13 (18.1)	
Antipsychotic only	8 (13.1)	12 (16.7)	
Mood stabilizer + antipsychotic	32 (52.5)	35 (48.6)	
Antipsychotic + antidepressant	5 (8.2)	6 (8.3)	
Mood stabilizer + antipsychotic + antidepressant	7 (11.5)	6 (8.3)	

Values are presented as the median (IQR) or *n* (%), as appropriate. Normality of continuous variables was assessed using the Shapiro–Wilk test. Continuous variables were compared using the independent samples *t*-test or the Mann–Whitney U test, depending on data distribution. Categorical variables were compared using the chi-square test. Abbreviations: IQR, interquartile range; ECT, electroconvulsive therapy; rTMS, repetitive transcranial magnetic stimulation.

**Table 2:** Comparison of functioning, internalized stigma, impulsivity, and anxiety measures between the subsyndromal depressive group and euthymic groups.

	Subsyndromal Depressive Group (*n* = 61)	Euthymic (*n* = 72)	*p*
HDRS total score, median (IQR)	10 (8–12)	3 (1–4)	<0.001
BDFS total score, mean ± SD	100.42 ± 15.19	113.62 ± 14.21	<0.001
BDFS—sexual functioning	7 (5–10)	8 (7–11)	0.008
BDFS–domestic relationships	14 (11–16)	14 (12–17)	0.047
BDFS–emotional functioning	7 (5–9)	9 (7–9)	<0.001
BDFS–cognitive functioning	9 (8–11)	11 (9–12)	0.005
BDFS–stigma	6 (5–8)	10 (7–12)	<0.001
BDFS–social withdrawal	6 (5–8)	8 (6–9)	<0.001
BDFS–relations with friends	11 (8–13)	11 (9–13)	0.205
BDFS–participation in social activities	12 (9–13)	12 (9–14)	0.307
BDFS–daily activities and hobbies	11 (10–13)	12 (11–14)	0.067
BDFS–initiative/potential use	6 (4–9)	7 (5–9)	0.072
BDFS–occupational functioning	11 (10–12)	11 (10–12)	0.220
ISMI total score	40 (30–48)	21 (15–34)	<0.001
ISMI–alienation	15 (12–18)	9 (8–12)	<0.001
ISMI–stereotype endorsement	13 (10–17)	10 (8–13)	<0.001
ISMI–perceived discrimination	12 (8–14)	8 (6–11)	<0.001
ISMI–social withdrawal	15 (11–18)	9 (8–13)	<0.001
ISMI–stigma resistance	14 (12–18)	15 (12–18)	0.185
BIS-11 total score	66 (54–72)	55 (45–67)	<0.001
BIS-11 attentional impulsivity	16 (13–19)	12 (10–16)	<0.001
BIS-11 motor impulsivity	20 (17–25)	18 (15–20)	0.013
BIS-11 non-planning impulsivity, mean ± SD	27.92 ± 4.94	24.81 ± 5.12	0.010
BAI total score	11 (6–24)	4 (1–8)	<0.001

Values are presented as the mean ± SD or median (IQR), depending on data distribution. Normality of continuous variables was assessed using the Shapiro–Wilk test. Continuous variables were compared using the independent samples *t*-test or the Mann–Whitney U test, depending on data distribution. Abbreviations: IQR, interquartile range; SD, standard deviation; HDRS, Hamilton Depression Rating Scale; BDFS, Bipolar Disorder Functioning Scale; BIS-11, Barratt Impulsiveness Scale; ISMI, Internalized Stigma of Mental Illness Scale; BAI, Beck Anxiety Inventory.

**Table 3:** Spearman correlations between HDRS total score and clinical, functioning, impulsivity, internalized stigma, and anxiety measures in the subsyndromal depressive group.

Variable	r	*p*
Clinical Characteristics		
Age at onset	0.121	0.339
Illness duration	0.082	0.503
Total number of episodes	0.194	0.125
Number of depressive episodes	0.281	0.020
Lifetime number of hospitalizations	−0.056	0.711
Psychometric Measures		
BDFS (total and subdomains)		
BDFS total score	−0.151	0.282
Emotional functioning	−0.061	0.630
Cognitive functioning	−0.161	0.182
Sexual functioning	−0.341	0.005
Stigma	−0.165	0.176
Social withdrawal	−0.132	0.289
Domestic relationships	−0.289	0.017
Relations with friends	−0.087	0.511
Participation in social activities	−0.116	0.351
Daily activities and hobbies	0.067	0.643
Initiative/potential use	0.083	0.539
Occupational functioning	−0.243	0.090
BIS-11 (total and subdomains)		
BIS-11 total score	0.237	0.055
Attentional impulsivity	0.315	0.009
Motor impulsivity	0.156	0.195
Non-planning impulsivity	0.160	0.185
ISMI (total and subdomains)		
ISMI total score	0.142	0.255
Alienation	0.249	0.047
Stereotype endorsement	0.114	0.368
Perceived discrimination	0.119	0.351
Social withdrawal	0.160	0.184
Stigma resistance	0.021	0.875
BAI total score	0.465	<0.001

Spearman’s correlation coefficient was used for correlation analyses. Statistical significance was set at *p* < 0.05. Abbreviations: HDRS, Hamilton Depression Rating Scale; BDFS, Bipolar Disorder Functioning Scale; BIS-11, Barratt Impulsiveness Scale; ISMI, Internalized Stigma of Mental Illness Scale; BAI, Beck Anxiety Inventory.

## Data Availability

The data presented in this study are available from the corresponding author upon reasonable request. The data are not publicly available due to privacy and ethical restrictions.
